# Supplementary material for: Ancestral function of Inhibitors-of-kappaB regulates Caenorhabditis elegans development
Source: Sci Rep. 2020 Sep 30;10:16153. doi: 10.1038/s41598-020-73146-5 (PMC7527347; doi:10.1038/s41598-020-73146-5)
Supplement: Supplementary file 1 — Supplementary Legends. [file 41598_2020_73146_MOESM1_ESM.docx]

**Supplementary Figure legends**

**Figure S1. Characterization of NFKI-1 and IKB-1. Related to Figure 1. (A, B)** Phylogenetic trees for NFKI-1 (A) and IKB-1 (B) proteins. The alignments were 900 and 2305 amino acids long, and presented on average a 52% and 39% of conservation, respectively^42^. **(C, D)** Potential SUMOylated lysines (k, shown in green) and SUMO-interaction sites (in red) in the NFKI-1 (C) and IKB-1 (D) proteins, as predicted using the GPS-SUMO tool^18^.

­

**Figure S2. Expression profiles of NFKI-1 and IKB-1. Related to Figure 1. (A)** RNA-seq time series^20,22^ showing *nfki-1* (green) and *ikb-1* (red) stage-specific expression profiles of embryonic development and all postembryonic stages. Expression data is presented as the average depth of coverage per million mapped reads (DCPM). Data were retrieved from GExplore 1.4 (www.genome.sfu.ca/gexplore)^21^. Plot was generated using Graphpad Prism 8. **(B)** Microscopy image of representative CER147 [*cerEx35[nfki-1p::nfki-1::TY1::EGFP::3xFLAG+unc-119(+)*] overexpression line at L4 larvae stage. Scale bar: 100 μm. The inset shows the boxed area at higher magnification. Inset scale bar: 10 μm. **(C)** Representative fluorescence microscopy images of a compound IKB-1::mCHERRY and GFP::MEL-28 L2 animal. IKB-1::mCHERRY display dot-like structure signals in the body wall muscle and pharynx tissues. GFP::MEL-28 endogenous reporter was used as a nuclear envelope marker. DAPI channel was merged to show gut autofluorescence. Scale bars: 50 μm.

**Figure S3. Endogenous reporters’ expression under starvation states and whole CDS deletion allele characterization. Related to Figure 2.** (**A**) Schematic representation of whole gene deletion mutant strain CER561 [*nfki-1(cer163[nfki-1p::EGFP1-3]) X]* by CRISPR-Cas9. Generation included the use of 2 crRNAs, one near the start codon (1, in orange) and the other near the stop codon (2, in purple). Protospacer adjacent motif (PAM) sequences are depicted in blue and underlined in the sequences. 35 bp-homology arms are highlighted in yellow. Chevron signs indicate *nfki-1*’s CDS last 23 nucleotides, we inserted an additional nucleotide to ensure the preservation of the frame (highlighted in pink). Black arrowheads depict the Cas9 cut site and black arrows show the insertion/deletion site. Full sequence of the repair template is displayed at the bottom. We included the flaking regions of an EGFP in the repair template (termed EGFP1 and EGFP3, respectively, highlighted in green), to facilitate a possible generation of a transcriptional reporter by Nested CRISPR methodology^49^. **(B)** Representative DIC images showing morphological defects observed in *nfki-1(cer163)* mutant animals. Scale bars: 20 μm. The graph indicates the percent of larvae with aberrant morphology, which is similar to the percentage observed with *nfki-1(cer2)* allele. *n>3500.* N=1. Error bars show upper and lower limits of 95% confidence intervals (CI) calculated by Wilson/Brown method of 1 experiment. Statistically significant differences were calculated using two-sided Chi-square test (CI: 95%). Plot was generated using Graphpad Prism 8.

**Figure S4. RNA-seqs analyses and H3K27me3 amount in IκB mutants. Related to Figures 3 and 4. (A)** Doughnut charts showing the distribution of genes categorized according to whether their expression is ubiquitous (green), germline-enriched (dark blue), germline-specific (light blue), soma-specific (yellow) or unclassified (gray) for differentially expressed genes at L1 stage. Numbers in the center represent the number of genes in each dataset. Categories dataset was extracted from^44^. Statistically significant differences between expected and observed distribution was calculated using Chi-square test for goodness of fit (p<0.05). Doughnut charts were generated using Graphpad Prism 8. **(B)** 2D plot illustrating the correlation between genes differentially expressed in *nfki-1* and *ikb-1* deficient animals at L1 stage. **(C)** PCA analysis of RNA-seq samples at L4 (orange) and L1 (blue) for wildtype (WT) and *nfki-1* and *ikb-1* mutants. ‘R’ denotes each replicate. **(D)** Western blot analysis of total protein lysates of L4 worms with the indicated genotypes using an H3K27me3 antibody. Histone H3 is used as a control. Uncropped images of the blots are included in **Supplementary Figure S5**. Plots in panels B and C were done with ggplot2 package (version 3.2.1, R software version 3.6.1).

**Figure S5. Uncropped images corresponding to blots and gels presented in the main Figures.**

**Supplementary tables.**

**Table S1. ChIP-seq analyses.** List of peaks and annotations of 3xFLAG::NFKI-1 and IKB-1::mCHERRY ChIP-seq at L1 stage. List of peaks and ChIP-seq annotation of H3K27me3 and H3K36me3 marks in *nfki-1* and *ikb-1* mutants.

**Table S2. RNA-seq analyses.** List of differentially expressed genes of IκB mutants’ RNA-seq at L4 stage. Classification of DEGs according to^44^.

**Table S3. List of strains used in this study.**

**Table S4. Primers sequences and CRISPR reagents used in this study.**
