# Supplementary material for: Ancestral function of Inhibitors-of-kappaB regulates Caenorhabditis elegans development
Source: Sci Rep. 2020 Sep 30;10:16153. doi: 10.1038/s41598-020-73146-5 (PMC7527347; doi:10.1038/s41598-020-73146-5)

Supplementary Information

Ancestral function of Inhibitors-of-kappaB regulates *Caenorhabditis elegans* development

David Brena, Joan Bertrán, Montserrat Porta-de-la-Riva, Yolanda Guillén, Eric Cornes, Dmytro Kukhtar, Lluís Campos-Vicens, Lierni Fernández, Irene Pecharromán, Albert Garcia-López , Abul B.M.M.K Islam , Laura Marruecos, Anna Bigas, Julián Cerón and Lluís Espinosa

Figure S1. Related to Figure 1.

A

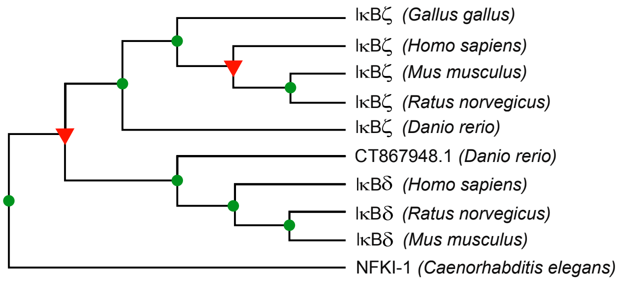

B

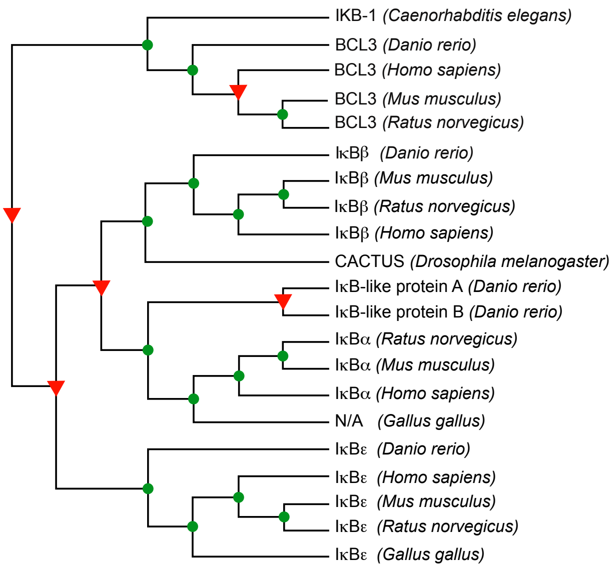

C

NFKI-1

```
  1 matvapkgnc lvaraipsds hadqltdllc klsvngdanq ksvnkfesqh gvtpsdfrdi
 61 qnirssalak ktktskfqld gvtlfadltp nskskkkten qetkekdeea eekkdgppkd
121 dkelkmkkek eqedenaeld eqkkdgdllg rgpvhnvrva tggshpyhra qipygcaaqt
181 pitdisaytg ygsgyecgst wslspdttig sisasttpdt vlssdgygsa sppqhspkes
241 lqspfsdiss adtsrvltpe nnelpeslqd filqysnqyt keesirgrpp sadsgvsspm
301 sarsapyasp hvpqgtcsgp ttpsfnqtrl sprtsengit akqrlnaiip esdlatgfhw
361 acttwknvlt nrdadgdtpl hivaahndlg kiyalcetlr ktmnenddnv fnvsnnfget
421 plyvavlqrs ievveyllel gaspnsrssr avgdsplhfa targmnnmve allskreirv
481 netnddgqts llcavkmhgm mdeqtqhkid nksiiemlik agadptiaet stgktivhha
541 vdkmdvelld flktvvnedt ftelanlsdf hgdtavdllc sstqnedtnn vrenlyirll
601 tsgavpnksr a
```

D

IKB-1

```
  1 mskadendan ltengvhksr sdseinshts sihksssqhr rlfiddisti easagdrvwa
 61 tcsgagdate litihflgdq feesaidvvr khslvmftvp slkvdselev elrfstsndn
121 vttisfkylp rkiqrshtqe islfdnllef atngdtisll qpfvaqiskq diegntvfhv
181 aakngqsfsl klllsvippd iknevinvqn thgltalhva irtgdpdavh ylmnhgakid
241 isdnhgstal hylgdaynes ifkeilepsr gqrfdvnqln segfapihva vrrlklslie
301 mlieagalid fldtekkrna lmhaiemndf etiqllverg sgtniedesg etalslavkn
361 vnypviglll dngadpnrqn skgicladse dsviqniing drpelpkkea fgvpndlais
421 rsplfgrshp dqapgeesgr hrvvkrsree ilndaqtlla etdsmaprvs ristsesede
481 qqpgpstssg trrrrsewdr npklsdsvcn ldyltrirva kifddqckwq rlaqqlncdh
541 mvelisicss gddssptmil ldqfeqleds sisrlrdamn qmaeddavkl idsryvy
```

Figure S2. Related to Figure 1.

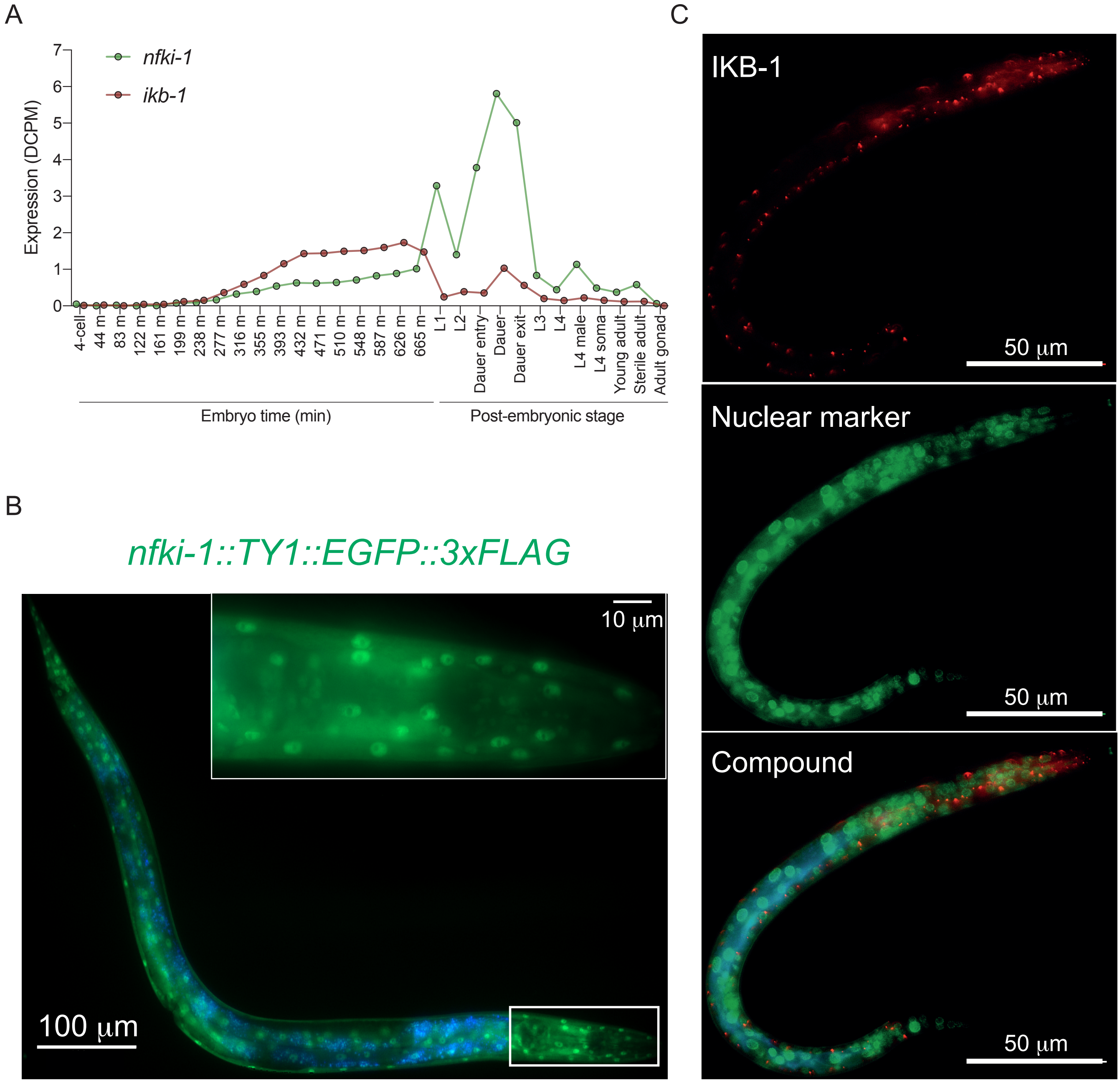

Figure S3. Related to Figure 2.

A

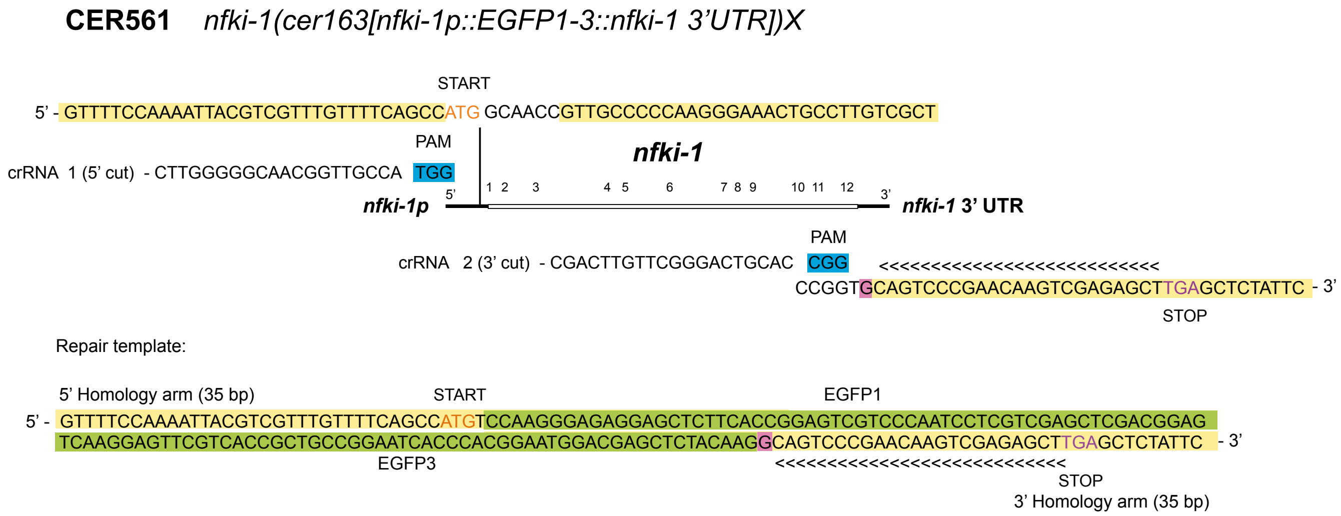

B

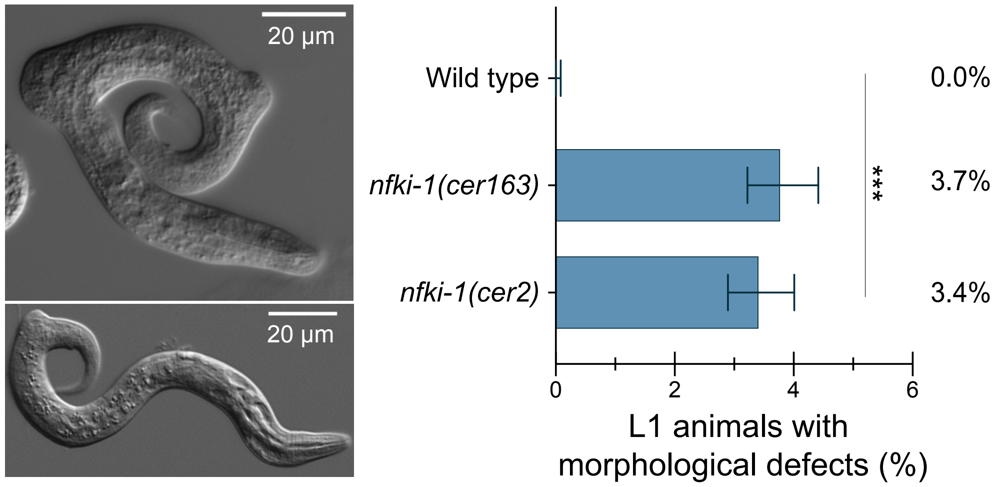

Figure S4. Related to Figure 4.

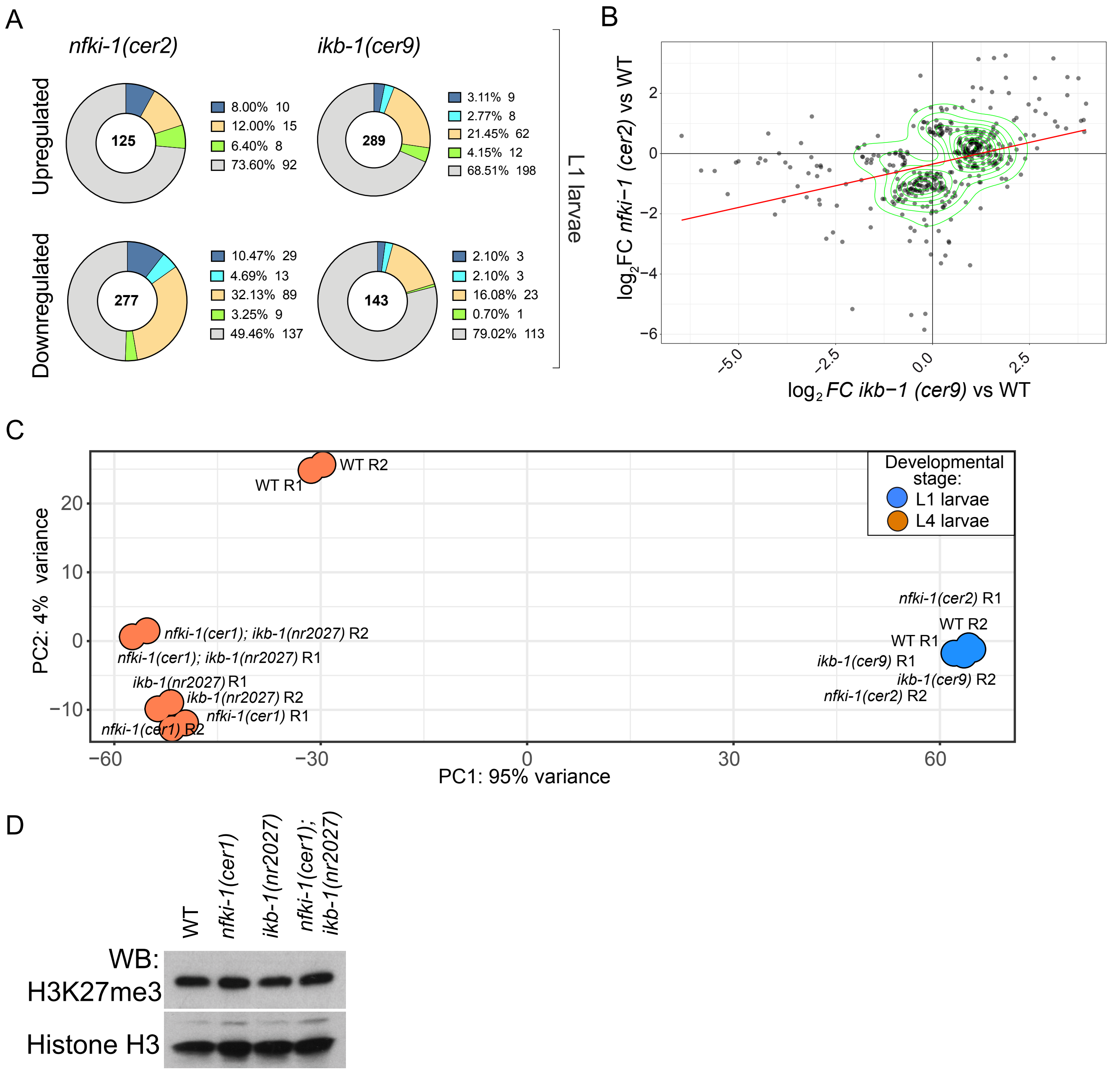

Figure S5.

Figure 1D

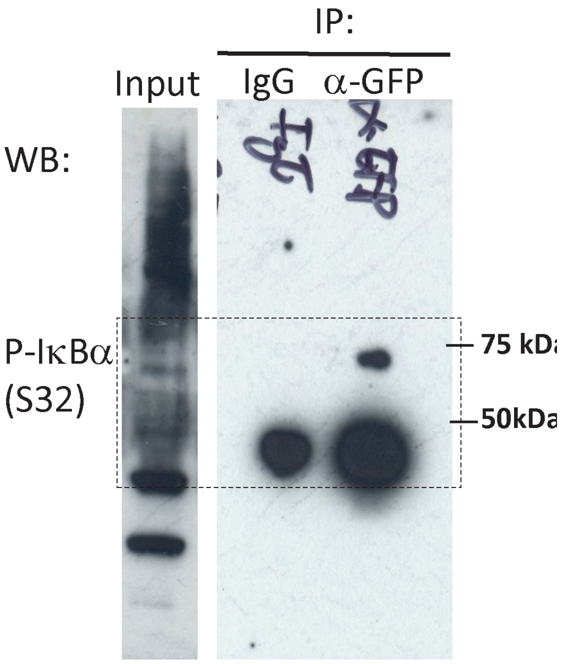

Figure 3A

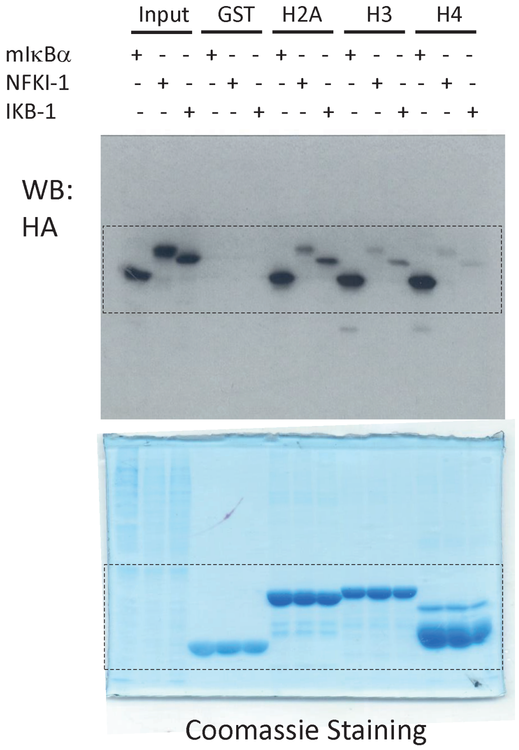

Figure 3B

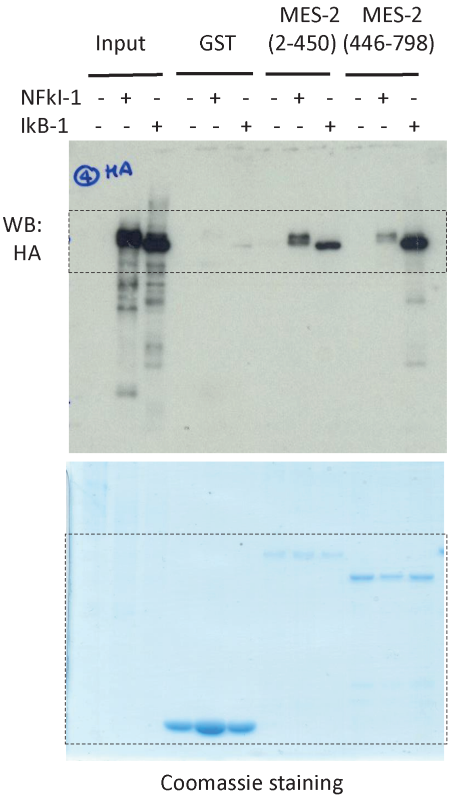

Figure 3C

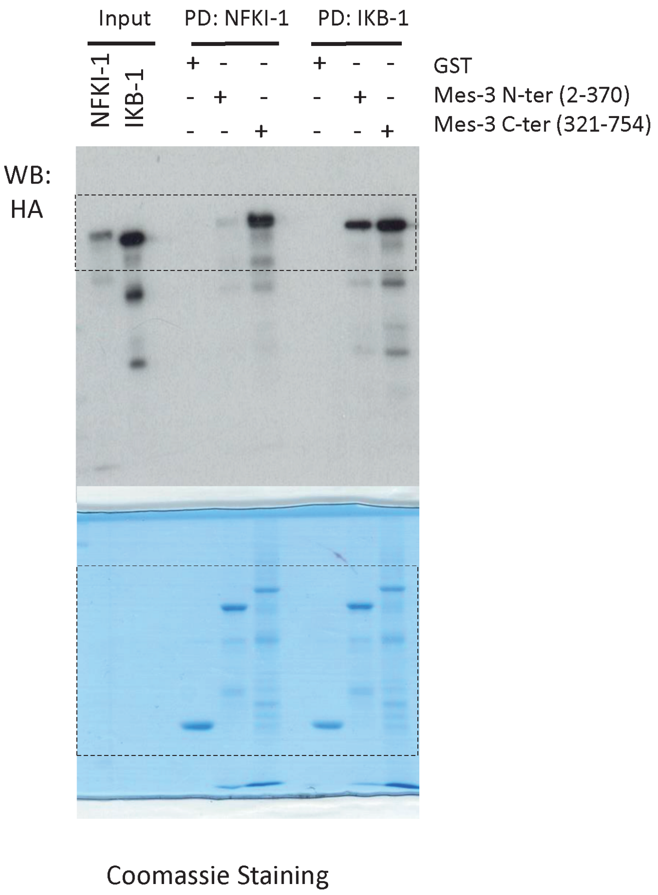

Figure 3D

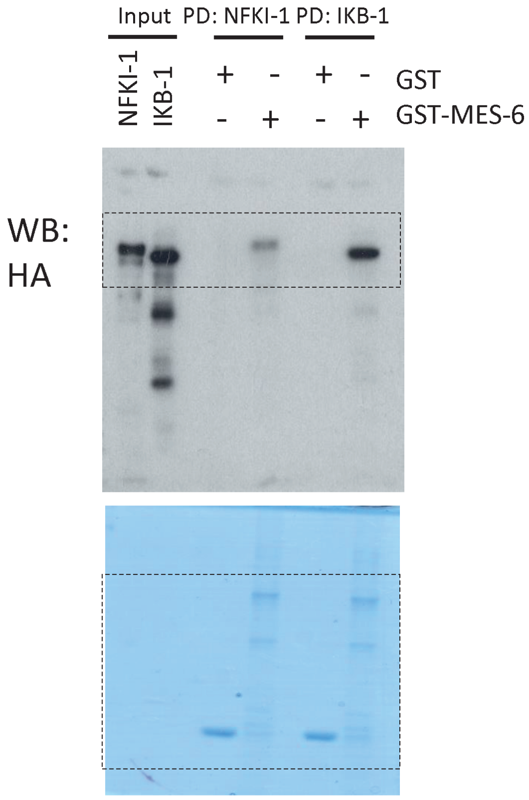

Figure 3F

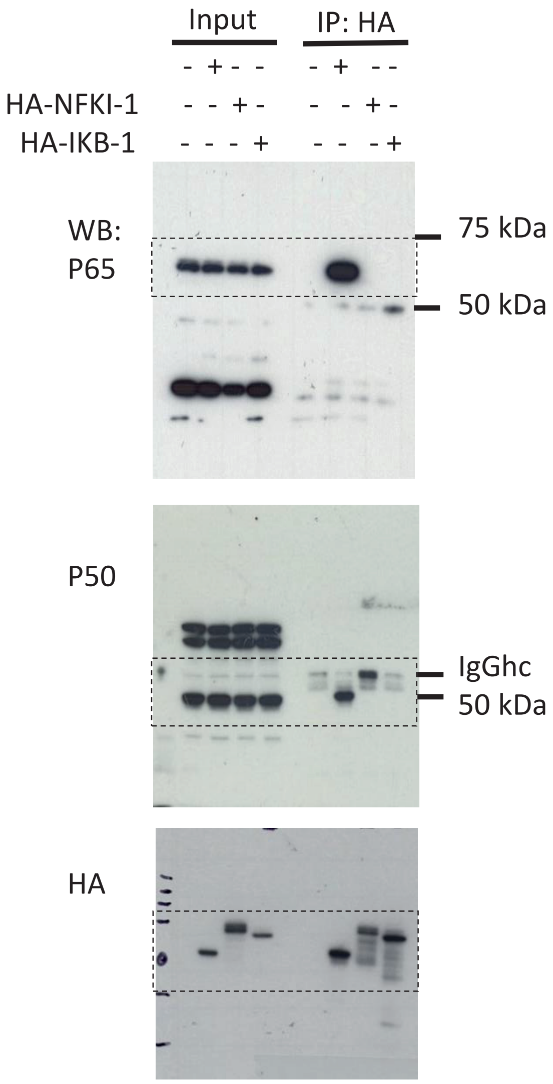

Figure S4D

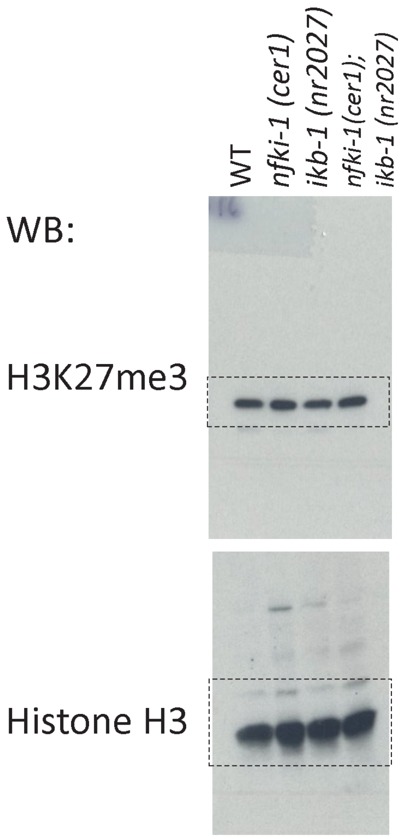

Supplement: Supplementary file 2 — Supplementary Figures. [file 41598_2020_73146_MOESM2_ESM.pdf]
